# Emotional Intelligence, Self-Regulation, and Children’s Well-Being in Fourth-Grade Students: Cross-Sectional Associations from Türkiye

**DOI:** 10.3390/jintelligence14060107

**Published:** 2026-06-11

**Authors:** Ümit İzgi Onbaşılı, Aliye Tekir, Feride Ercan Yalman

**Affiliations:** 1 Department of Elementary Education, Faculty of Education, Mersin University, 33110 Mersin, Türkiye; aliyetekir@gmail.com; 2 Department of Science Education, Faculty of Education, Mersin University, 33110 Mersin, Türkiye; feride@mersin.edu.tr

**Keywords:** children’s well-being, emotional intelligence, self-regulation, elementary school students, socio-emotional development, indirect association

## Abstract

This study examined the associations of self-reported emotional intelligence and self-regulation with children’s well-being among fourth-grade elementary school students in Mersin, Türkiye. The sample comprised 627 students, predominantly aged 9 to 10 years, from seven public elementary schools selected to reflect different district and school contexts. Data were collected in person after ethics committee approval, institutional permissions from the Turkish Ministry of National Education, and written parental consent. The Children’s Emotional Intelligence Scale, the Self-Regulation Scale, and the Stirling Children’s Well-Being Scale were administered. Descriptive statistics, Pearson correlations, simple and multiple linear regressions, and a cross-sectional indirect association analysis using PROCESS Model 4 with 5000 bootstrap resamples were conducted. Emotional intelligence was positively associated with children’s well-being and self-regulation, while self-regulation showed a weaker positive association with well-being. Emotional intelligence explained 31.4% of the variance in well-being, self-regulation explained 8.6% when examined alone, and both variables jointly explained 31.9%, indicating only a marginal increase over emotional intelligence alone. Thus, most of the explained variance was accounted for by emotional intelligence, whereas self-regulation made a very small incremental contribution beyond it. The indirect association analysis indicated a small but statistically supported pattern of indirect association between emotional intelligence and well-being through self-regulation within this cross-sectional design; the association between emotional intelligence and well-being remained significant after self-regulation was included in the model. The findings suggest that emotional intelligence is the stronger socio-emotional correlate of children’s well-being in this sample, whereas self-regulation shows a limited complementary association. Given the cross-sectional design and reliance on self-report measures, the findings should be interpreted as correlational associations rather than evidence of causal effects, temporal ordering, or developmental change. Future studies should use longitudinal, intervention-based, and multi-informant designs to further examine these associations.

## 1. Introduction

Children’s well-being is considered a key developmental outcome closely associated with emotional, social, and academic development during the elementary school years (Keyes et al., 2002; Ryff, 1989; Ryff & Singer, 2008). During this period, children’s peer relationships, classroom responsibilities, emotional awareness, and behavioral regulation become increasingly important, making well-being relevant not only to school adjustment but also to broader socio-emotional functioning (Dülgergil, 2024; Kaya Memiş, 2018). The positive psychology approach similarly positions well-being as an important educational aim by emphasizing students’ strengths and their application in school contexts (Seligman et al., 2009). In the present study, children’s well-being is considered within the elementary school context as a broad indicator of emotional and psychological functioning.

Contemporary educational policies likewise address student well-being as a complementary dimension of quality education. The European Commission’s framework emphasizes that schools should serve as safe developmental environments supporting students’ mental health, psychosocial skills, and learning processes (European Commission, 2024), and a recent guide published on the European School Education Platform highlights that school well-being can be strengthened through a safe climate, respectful relationships, social and emotional learning, student participation, and accessible mental health support (European School Education Platform, 2025). OECD reports further show that social and emotional skills are associated with life satisfaction, psychological well-being, academic achievement, and healthy behaviors (OECD, 2021, 2024). The Sustainable Development Goals related to Good Health and Well-Being and Quality Education similarly indicate that children’s psychological health and educational development should be addressed together (UNDP, 2015a, 2015b), while UNESCO’s vision for the futures of education positions education as a transformative process that supports individual and collective well-being rather than limiting it to the transmission of academic knowledge (UNESCO, 2021).

Emotional intelligence is one of the socio-emotional constructs that may be associated with children’s well-being. In the present study, it is treated as self-reported emotional intelligence, reflecting children’s reported emotional awareness, empathy, motivation, and emotion management. Previous studies indicate that emotional intelligence is associated with well-being, social adjustment, and school experiences in children and adolescents (Gao et al., 2024; Guerra-Bustamante et al., 2019; Llamas-Díaz et al., 2022; Molero et al., 2025). In this respect, emotional intelligence may be particularly relevant during the elementary school years, when children are expected to recognize their own emotions, respond to others’ emotions, manage peer relationships, and cope with emotional demands in school life.

Self-regulation is another developmental resource relevant to school functioning and well-being. It involves children’s capacity to manage attention, effort, learning strategies, motivation, and behavior in goal-directed ways (Maes & Gebhardt, 2000; B. J. Zimmerman, 2000). Previous research links self-regulation with academic functioning, developmental adjustment, and well-being (Fomina et al., 2020; Rodríguez et al., 2022). Although emotional intelligence and self-regulation are conceptually related, they represent distinct developmental resources. Emotional intelligence mainly concerns children’s emotional and social functioning, whereas self-regulation is more closely related to the management of attention, effort, learning strategies, and goal-directed behavior. Because the present study is cross-sectional, the relationships among these constructs are examined as concurrent associations rather than as evidence of a causal developmental sequence.

In the existing literature, studies seeking to explain children’s well-being have addressed various individual and contextual variables, including emotional intelligence, emotional self-efficacy, coping, self-perception, parenting styles, and self-regulation (Fomina et al., 2020; Gao et al., 2024; Pauletto et al., 2021). These studies contribute to an understanding of well-being as associated with both emotional and self-regulatory resources. However, fewer studies have examined children’s emotional intelligence and self-regulation simultaneously within the same analytical model, particularly among children aged 9 to 10 years. This age range is developmentally meaningful because it falls within middle childhood, a period in which children’s self-understanding, peer comparison, achievement-related beliefs, school-related responsibilities, and relationships beyond the family become increasingly salient (Eccles, 1999). In this developmental period, examining emotional and self-regulatory resources together is theoretically relevant because children are expected to manage emotional experiences, interact with peers, sustain attention and effort, and respond to increasing academic demands in school contexts. Therefore, the simultaneous examination of emotional intelligence and self-regulation allows the present study to evaluate their relative and complementary associations with children’s well-being and to examine whether the association between emotional intelligence and well-being includes an indirect association through self-regulation.

The present study addresses this gap by examining the associations among self-reported emotional intelligence, self-regulation, and children’s well-being in a sample of fourth-grade elementary school students, predominantly aged 9 to 10 years, in Türkiye. The study contributes to the literature in three respects. First, it provides evidence from a relatively large sample of 9- to 10-year-old fourth-grade students in the Turkish elementary school context, where integrated evidence on these constructs remains limited. Second, it examines emotional intelligence and self-regulation within the same analytical model rather than treating them in isolation. Third, it evaluates whether self-regulation represents a statistically supported indirect association between emotional intelligence and well-being, while interpreting this pattern cautiously within a cross-sectional design. Given the cross-sectional nature of the study, any indirect association is interpreted as a relational pattern rather than as evidence of a causal mechanism. In this respect, the study aims to offer a more differentiated understanding of how emotional and self-regulatory resources are related to children’s well-being in the elementary school context.

### 1.1. Theoretical Background: Children’s Well-Being, Emotional Intelligence, and Self-Regulation

In the present study, children’s well-being is interpreted in line with the conceptual scope of the Stirling Children’s Well-Being Scale, which focuses on children’s emotional and psychological functioning rather than on a single indicator of happiness or school satisfaction (Akın et al., 2016; Liddle & Carter, 2015). This framing is consistent with the broader distinction between hedonic and eudaimonic approaches to well-being. Subjective well-being is generally associated with the hedonic tradition and refers to individuals’ cognitive and emotional evaluations of their lives, including life satisfaction, positive affect, and reduced negative affect (Diener, 1984; Diener et al., 1999). Emotional well-being is closely related to this affective dimension and emphasizes the presence of positive emotional experiences and the reduction in negative ones (Diener et al., 1999). Psychological well-being, by contrast, is grounded in the eudaimonic tradition and refers to positive psychological functioning, meaningful goals, personal growth, positive relationships, and effective engagement with one’s environment (Ryan & Deci, 2001; Waterman, 1993). Ryff’s framework similarly shows that well-being is not limited to positive emotional states but also includes self-acceptance, positive relations with others, autonomy, environmental mastery, personal growth, and purpose in life (Ryff, 1989; Ryff & Singer, 2008). Although hedonic and eudaimonic approaches are conceptually distinct, they provide complementary perspectives for understanding children’s functioning in school contexts (Karaduman Şermet, 2024). Accordingly, well-being in this study is conceptualized as a holistic outcome reflecting children’s emotional and psychological functioning within the elementary school context.

This holistic understanding is particularly relevant to the school context. Conceptual models of school well-being emphasize that children’s well-being should be considered together with relationships, learning conditions, health, belonging, and self-actualization processes within the school environment (Hascher, 2008; Konu & Rimpelä, 2002). School is therefore not only a setting in which well-being is observed but also a developmental environment in which emotional, social, academic, and self-management processes are shaped. During the elementary school years, children encounter increasing academic responsibilities, structured classroom routines, peer relationships, and expectations for behavioral regulation. For this reason, children’s well-being should be examined together with the emotional and regulatory resources that support functioning in everyday school life (OECD, 2021, 2024; Seligman et al., 2009). In the Turkish elementary school literature, children’s well-being has also been examined in relation to perceived school kindness, mindfulness-based practices, friendship relations, and broader conceptualizations of elementary school students’ well-being (Alpay, 2021; Dülgergil & Çelik, 2023; Erol, 2022; Kaya Memiş, 2018). These studies collectively suggest that children’s well-being is a school-related developmental construct shaped by relational, emotional, and self-regulatory processes.

A central conceptual issue in the present study concerns how emotional intelligence is understood and measured. Emotional intelligence has been discussed through different theoretical traditions, including ability-based, trait-based, and mixed-model approaches (Bru-Luna et al., 2021; Pérez et al., 2005). Ability-based models define emotional intelligence as a set of cognitive-emotional abilities related to perceiving, using, understanding, and managing emotions (Mayer & Salovey, 1997; Salovey & Mayer, 1990). Trait-based approaches emphasize individuals’ self-perceptions of emotional functioning and typical emotional responses, generally measured through self-report inventories (Petrides & Furnham, 2001; Petrides et al., 2007). Mixed models adopt a broader perspective by considering emotional and social dimensions together, such as emotional awareness, motivation, empathy, emotion management, and social functioning (Goleman, 1995). This distinction is important because the Children’s Emotional Intelligence Scale used in the present study is neither a performance-based ability test nor a trait emotional intelligence instrument such as the TEIQue. Rather, it is a child self-report measure assessing emotional awareness, empathy, motivation, and emotion management (Küçükkaragöz & Kocabaş, 2012). Therefore, emotional intelligence is retained as the scale and variable label throughout the manuscript, but it is interpreted as self-reported emotional intelligence within a mixed-model-oriented educational framework.

This conceptualization is consistent with the developmental and educational focus of the study. For children, self-reported emotional intelligence may be reflected in how they recognize their own emotions, understand others’ emotions, sustain motivation, manage emotional reactions, and participate in peer and teacher relationships. Earlier literature on emotional intelligence emphasized that cognitive abilities alone are not sufficient to explain students’ educational and personal development and that social and emotional dimensions are relevant to adjustment, relationship-building, and well-being processes (Çakar & Arbak, 2004). Recent research with children and adolescents similarly shows that emotional intelligence is associated with well-being, perceived happiness, social adjustment, and school experiences (Gao et al., 2024; Guerra-Bustamante et al., 2019; Llamas-Díaz et al., 2022). Research on emotional intelligence in children further indicates that emotional awareness, interpersonal relationships, school life, and socio-emotional functioning are closely connected to children’s developmental experiences (Özal et al., 2024). Related evidence on trait emotional intelligence in childhood also suggests that children’s self-reported emotional characteristics are associated with psychological well-being and may offer additional insight into developmental adjustment beyond general cognitive ability (Pauletto et al., 2021). Intervention-based studies with elementary school students also support the view that emotional education and emotional intelligence can be considered together with children’s emotional well-being (Molero et al., 2025). Studies conducted in Türkiye are consistent with this tendency, reporting significant relationships between emotional intelligence and well-being in different child and adolescent samples (Çelik, 2008; Güngör, 2023). Taken together, these findings support the relevance of self-reported emotional intelligence as a socio-emotional resource associated with children’s well-being.

Self-regulation represents a second developmental resource in the model. Self-regulation refers to the processes through which individuals initiate, monitor, sustain, and reorganize their emotions, thoughts, motivation, and behaviors in line with their goals (B. J. Zimmerman, 2000; B. Zimmerman, 2002). In classroom learning, self-regulation involves planning, goal setting, attention management, effort regulation, strategy use, help seeking, and self-evaluation (Boekaerts et al., 2000; Paris & Paris, 2001). In this respect, self-regulation is not only an academic learning skill but also a broader self-management capacity that supports children’s behavioral adjustment, motivation, and developmental functioning in school life. During the elementary school years, students are increasingly expected to direct their attention to tasks, sustain effort, use learning strategies, seek support when necessary, and evaluate their own performance (Dignath et al., 2008; Maes & Gebhardt, 2000; Paris & Paris, 2001; Sarı & Akınoğlu, 2013; B. J. Zimmerman, 2000; B. Zimmerman, 2002). Self-regulation is therefore theoretically relevant to children’s well-being because it reflects how children manage the cognitive, emotional, motivational, and behavioral demands of school life.

The relationship between self-regulation and well-being is supported by international and Turkish research. Longitudinal findings suggest that children’s self-regulatory capacity is related to later well-being and learning experiences (Chu et al., 2020; Fomina et al., 2020). Systematic review evidence also indicates that self-regulatory strategies, executive functions, and regulatory skills are connected to different dimensions of student well-being (Rodríguez et al., 2022). In Türkiye, self-regulation skills have been linked to academic achievement among fourth-grade students (Yılmaz, 2020) and to social-emotional well-being and psychological resilience among preschool children (Özbey et al., 2022). These findings suggest that self-regulation should be considered not only as a learning-related construct but also as a developmental resource associated with children’s psychological and social functioning.

The theoretical connection between self-reported emotional intelligence and self-regulation provides the basis for examining these constructs together. Self-reported emotional intelligence reflects children’s reports of emotional awareness, empathy, motivation, and emotion management, whereas self-regulation reflects the goal-directed management of attention, effort, learning strategies, motivation, and behavior. These constructs are related because children who report stronger emotional intelligence may also report stronger self-management capacities in school life. At the same time, they remain conceptually distinct. Self-reported emotional intelligence mainly represents the emotional and social side of children’s functioning, whereas self-regulation represents the goal-directed management of learning, effort, behavior, and self-monitoring. Existing findings support this distinction by showing that emotional intelligence is associated with emotional well-being, motivation, and learning strategies, while self-regulatory processes are linked to learning responsibility, strategy use, and academic functioning (Nieto-Carracedo et al., 2024; Paris & Paris, 2001; Schlesier et al., 2019). Studies conducted at different educational levels in Türkiye also suggest that emotional intelligence and self-regulation can be examined together within a developmental framework (Aydın, 2018; Sarıkan, 2023; Yalçın, 2023).

In the present model, well-being is treated as the outcome reflecting children’s emotional and psychological functioning. Self-reported emotional intelligence is positioned as a socio-emotional resource related to emotional awareness, empathy, motivation, and emotion management, whereas self-regulation is positioned as a self-management resource related to attention, effort, learning strategies, goal orientation, and behavioral monitoring. From a theoretical perspective, examining these constructs within the same model makes it possible to investigate how emotional and self-regulatory resources are associated with children’s well-being, whether self-regulation explains additional variance beyond emotional intelligence, and whether the association between emotional intelligence and well-being includes a statistically supported indirect association through self-regulation. This approach provides an analytical basis for evaluating the relative and complementary associations of emotional intelligence and self-regulation with children’s well-being. Because the data are cross-sectional, any indirect association through self-regulation is not interpreted as evidence of a causal mechanism, developmental sequence, or temporal ordering. Instead, it is treated as a theoretically informed relational pattern among the variables.

### 1.2. Aim of the Study

The aim of this study is to examine children’s well-being in relation to emotional intelligence and self-regulation among fourth-grade elementary school students. In the study, well-being was treated as the outcome variable reflecting children’s emotional and psychological functioning. Emotional intelligence was examined as self-reported emotional intelligence within a mixed-model-oriented framework, whereas self-regulation was examined as a self-management resource related to children’s goal-directed functioning in school contexts. Accordingly, the study aimed to describe students’ levels of emotional intelligence, self-regulation, and well-being; determine the relationships among these variables; examine the variance in well-being accounted for by emotional intelligence and self-regulation when considered separately and jointly; and examine whether emotional intelligence is indirectly associated with well-being through self-regulation within a cross-sectional model. Because the study was cross-sectional, this indirect association was interpreted as a relational pattern rather than as evidence of a causal mechanism, developmental sequence, or temporal ordering. In this respect, the study seeks to contribute to a more differentiated understanding of how emotional and self-regulatory resources are related to children’s well-being in elementary school contexts.

### 1.3. Objectives and Hypotheses

This study was structured as a cross-sectional correlational study examining the concurrent relationships among emotional intelligence, self-regulation, and well-being in fourth-grade elementary school students. In line with the theoretical framework, the study focused on bivariate associations, variance accounted for in well-being, and a theoretically informed indirect association through self-regulation. Accordingly, the study sought to answer the following research questions:

RQ1. What are fourth-grade elementary school students’ levels of emotional intelligence, self-regulation, and well-being?
RQ2. What are the relationships among emotional intelligence, self-regulation, and well-being?
RQ3. How much variance in children’s well-being is accounted for by emotional intelligence and self-regulation when they are examined separately and jointly?
RQ4. Does the association between emotional intelligence and children’s well-being include an indirect association through self-regulation?

#### Hypotheses

Based on the theoretical framework, the following hypotheses were formulated:

**H1.**  *Emotional intelligence, self-regulation, and well-being are expected to be positively interrelated.*

**H2.**  *Emotional intelligence and self-regulation are expected to be positively associated with children’s well-being when examined separately and together.*

**H3.**  *The association between emotional intelligence and children’s well-being is expected to include a positive indirect association through self-regulation.*

**Figure 1:**
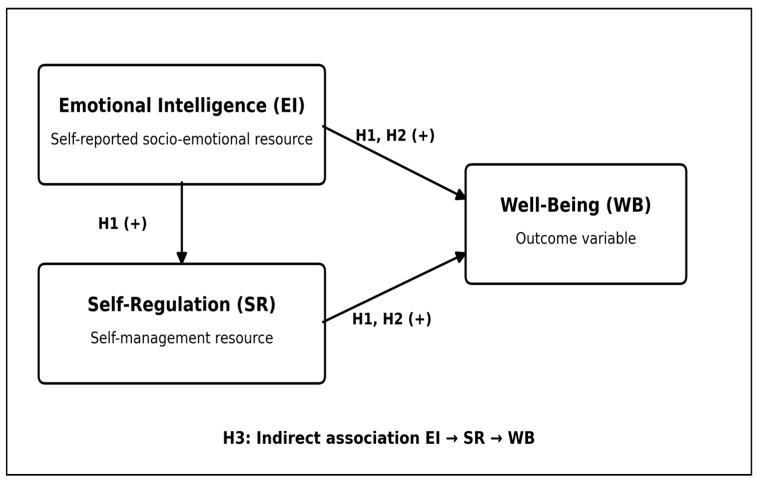
Conceptual model of the hypothesized associations among emotional intelligence, self-regulation, and children’s well-being. Note. EI = emotional intelligence; SR = self-regulation; WB = well-being.

As shown in Figure 1, the conceptual model summarizes the hypothesized associations among emotional intelligence, self-regulation, and well-being. Emotional intelligence is expected to be positively associated with both well-being and self-regulation, while self-regulation is expected to be positively associated with well-being. The model also examines whether the association between emotional intelligence and well-being includes an indirect association through self-regulation. Given the cross-sectional and correlational nature of the study, this model is interpreted as a theoretically informed relational pattern rather than as evidence of causal or temporal ordering.

## 2. Materials and Methods

This study was designed using a quantitative research approach to examine the relationships among emotional intelligence, self-regulation, and well-being in elementary school students. A cross-sectional correlational research design was employed. Correlational research designs are appropriate for examining the strength and patterns of association among variables without manipulating them (Cohen et al., 2018; Creswell & Creswell, 2018). Because the study was based on cross-sectional data, the findings were interpreted as concurrent associations, regression-based associations, and a theoretically grounded pattern of indirect association rather than as causal effects. Therefore, the indirect association estimates were not interpreted as evidence of a causal mechanism, developmental sequence, or temporal ordering but as statistical indirect associations based on cross-sectional data (Maxwell & Cole, 2007).

To address the possible role of demographic and family background characteristics, additional covariate-adjusted analyses were planned. Gender, nationality, number of siblings, family size, maternal education level, and paternal education level were considered as available covariates. These analyses were used as robustness checks to examine whether the main associations among emotional intelligence, self-regulation, and well-being remained stable after accounting for these characteristics.

### 2.1. Participants

The study group consisted of 627 fourth-grade students enrolled in public elementary schools affiliated with the Ministry of National Education in Mersin during the 2025–2026 academic year. In the Turkish education system, the fourth grade of elementary school generally corresponds to the 9 to 10 age group. In the study, purposive sampling, one of the non-probability sampling methods, was used, and a maximum variation strategy was adopted in school selection to enhance the contextual diversity of the sample. Accordingly, seven public elementary schools were identified in Mersin to reflect different district and school contexts, and the data collection process was carried out in these schools after obtaining the required ethics committee approval and institutional permissions from the Ministry of National Education.

In the selection of schools, attention was paid to reaching students from different settlement contexts, ensuring the feasibility of implementation, obtaining the necessary permissions, and working with school administrations that were open to collaboration. The purpose of this selection process was not to compare schools or districts, but to examine the relationships among emotional intelligence, self-regulation, and well-being in elementary school students across more diverse school contexts. For this reason, school and district were treated as contextual sampling characteristics rather than as substantive variables in the main analytical model. This decision was consistent with the sampling strategy because the schools were selected to increase contextual variation rather than to estimate school-level or district-level effects. Of the participants, 52.6% were girls and 47.4% were boys. In terms of nationality, the great majority of the students were citizens of the Republic of Türkiye (87.1%). Regarding the number of siblings, the highest proportion consisted of students who had one sibling (39.4%). When family size was examined, more than half of the students were found to live in families of five or more members (51.1%). In terms of parental education, primary school education constituted the largest category for both mothers and fathers. The proportion of mothers with university-level education or higher was 19.9%, while the corresponding proportion for fathers was 20.3%.

Basic demographic information about the participants was collected to describe the contextual characteristics of the sample and to transparently indicate the student profile through which the study findings should be interpreted. In addition to descriptive reporting, the available demographic and family background variables were used in covariate-adjusted robustness analyses. These variables included gender, nationality, number of siblings, family size, maternal education level, and paternal education level. For variables with missing responses, percentages were calculated based on the number of valid responses for each variable. Detailed demographic characteristics of the participants are presented in Appendix A, Table A1.

### 2.2. Measures

Data were collected using three scales. These scales were used to determine elementary school students’ levels of well-being, emotional intelligence, and self-regulation. The scales were selected because they were appropriate for child samples and had previously undergone validity and reliability studies. In line with the conceptual framework of the study, emotional intelligence was assessed as self-reported emotional intelligence rather than as performance-based ability emotional intelligence. In the present study, both total item scores and item mean scores were calculated for each scale. Considering the different numbers of items and response ranges across the variables, item mean scores were used in the correlation, regression, and indirect association analyses.

#### 2.2.1. Emotional Intelligence

The Children’s Emotional Intelligence Scale was used to determine students’ levels of emotional intelligence. The scale was developed by Küçükkaragöz and Kocabaş (2012) to assess children’s emotional intelligence. It consists of 18 items and four subdimensions. These subdimensions are emotional awareness, empathy, motivation, and managing emotions. The scale uses a four-point Likert-type response format, and total scores range from 18 to 72. Higher scores indicate higher levels of emotional intelligence. Because the scale is based on children’s self-reports and includes dimensions such as emotional awareness, empathy, motivation, and managing emotions, it was interpreted in this study as a measure of self-reported emotional intelligence within a mixed-model-oriented framework rather than as a performance-based ability measure. In the present study, the item mean score of the scale was used in the analyses. The Cronbach’s alpha coefficient of the emotional intelligence scale was 0.718, and the McDonald’s omega coefficient was 0.732 in the current sample.

#### 2.2.2. Self-Regulation

The Self-Regulation Scale was used to determine students’ levels of self-regulation. The scale was developed to assess the self-regulation skills of fourth-grade elementary school students (Yılmaz, 2020). It consists of 18 items and five subdimensions. These subdimensions are goal setting and planning, learning strategies, effort regulation, help seeking, and self-evaluation. The scale uses a three-point Likert-type response format, and total scores range from 18 to 54. Higher scores indicate higher levels of self-regulation skills. In the present study, the item mean score of the scale was used in the analyses. The Cronbach’s alpha coefficient of the self-regulation scale was 0.749, and the McDonald’s omega coefficient was 0.760 in the current sample.

#### 2.2.3. Children’s Well-Being

The Turkish form of the Stirling Children’s Emotional and Psychological Well-Being Scale was used to determine students’ levels of well-being. The original form of the scale was developed by Liddle and Carter (2015) to assess children’s emotional and psychological well-being in a holistic manner. The validity and reliability study of the Turkish form was conducted by Akın et al. (2016). The Turkish form consists of 12 items and uses a five-point Likert-type response format. The one-dimensional model of the Turkish form was reported to show acceptable fit. Total scores range from 12 to 60. Higher scores indicate higher levels of children’s emotional and psychological well-being. In the present study, the item mean score of the scale was used in the analyses. The Cronbach’s alpha coefficient of the well-being scale was 0.815, and the McDonald’s omega coefficient was 0.816 in the current sample.

### 2.3. Procedure

The data were collected from fourth-grade students enrolled in public elementary schools affiliated with the Ministry of National Education in Mersin during the 2025–2026 academic year. Before the data collection process, the required ethics committee approval was obtained. The study was approved as ethically appropriate by the Mersin University Educational Sciences Ethics Committee with the approval dated 7 October 2025, meeting number 11, and decision number 220. Following ethics committee approval, the necessary institutional permissions were obtained to conduct the study. The data collection process was carried out in schools affiliated with the Ministry of National Education at times deemed appropriate by school administrations and classroom teachers. Before implementation, school administrators, teachers, parents, and students were informed about the purpose of the study, the principle of voluntary participation, and the confidentiality of the data. Written parental consent was obtained for students to be included in the study, and students’ participation was based on voluntariness. The scales were administered to students face to face in the classroom environment. The implementation was conducted during a class period deemed appropriate by the school and the classroom teacher and was completed within approximately one class period. Students were informed that they should not write any identifying information while responding to the scales. They were also told that their responses would be used only for scientific purposes and evaluated in accordance with the principle of confidentiality. No personally identifying information was collected from students, and all analyses were conducted on anonymized data.

### 2.4. Validity Evidence and Internal Consistency Reliability

The scales used in the study were selected because they were appropriate for school-age child samples and had previously undergone scale development or adaptation studies reporting validity and reliability evidence (Akın et al., 2016; Küçükkaragöz & Kocabaş, 2012; Yılmaz, 2020). In the present study, validity evidence was considered on the basis of these previous studies, including published evidence regarding scale content, target populations, and reported dimensional structures. Internal consistency reliability was then examined in the current sample using Cronbach’s alpha and McDonald’s omega coefficients. The analysis results showed that the Cronbach’s alpha coefficient for the emotional intelligence scale was 0.718 and the McDonald’s omega coefficient was 0.732. For the self-regulation scale, the Cronbach’s alpha coefficient was 0.749 and the McDonald’s omega coefficient was 0.760. For the well-being scale, the Cronbach’s alpha coefficient was 0.815 and the McDonald’s omega coefficient was 0.816. These values indicate that the total scores of the scales used in the study had acceptable levels of internal consistency in the current sample. Because some subscale reliability coefficients were below acceptable levels, subscale scores were used only for descriptive purposes, and the total item mean scores were used in the main correlation, regression-based, and indirect association analyses.

### 2.5. Data Analysis

The data obtained in the study were analyzed using IBM SPSS Statistics 27.0 software (IBM Corp, 2020). Before the analyses, the dataset was examined, missing data were checked, and the analyses were conducted with participants who had complete data on the relevant variables. Because some demographic variables included missing responses, the number of valid cases differed across analyses involving these variables. Correlation, regression-based, and indirect association analyses were performed using the item mean scores obtained from the scales. Influential observations were evaluated using Cook’s Distance values. The low Cook’s Distance values indicated that there were no influential observations that could substantially affect the regression coefficients; therefore, the analyses were continued with the existing dataset. Within the scope of descriptive statistics, frequency, percentage, arithmetic mean, standard deviation, minimum, and maximum values were calculated. The internal consistency levels of the scales were evaluated using Cronbach’s alpha and McDonald’s omega coefficients.

Before conducting parametric analyses, the distributional characteristics of the variables were examined using skewness and kurtosis coefficients, histograms, Q-Q plots, and boxplots. Because of the large sample size, the assessment of normality was not based solely on significance tests; instead, distributional indicators and graphical examinations were considered together. Before the regression analyses, the assumptions of linearity, distribution of residuals, multicollinearity, and independence of error terms were checked. Multicollinearity was evaluated using VIF and tolerance values, while the independence of error terms was assessed using the Durbin–Watson coefficient. The findings obtained from the assumption checks indicated that the data were appropriate for correlation and regression-based analyses. The assumption and diagnostic check results for the main model are presented in Appendix A, Table A2. As an additional robustness check for the main multiple regression model, HC3 robust standard errors and 5000-resample bootstrap confidence intervals were also calculated. These results are presented in Appendix A, Table A3.

In line with the first research question, descriptive statistics were calculated for students’ levels of emotional intelligence, self-regulation, and well-being. Within the scope of the second research question, the relationships among the variables were analyzed using Pearson correlation coefficients. In line with the third research question, simple and multiple linear regression analyses were conducted to determine the variance in well-being accounted for by emotional intelligence and self-regulation when examined separately and jointly. In these analyses, well-being was treated as the outcome variable, while emotional intelligence and self-regulation were treated as explanatory variables in the regression models.

Additional covariate-adjusted models were conducted to examine whether the main associations remained stable after accounting for available demographic and family background characteristics. Gender, nationality, number of siblings, family size, maternal education level, and paternal education level were entered as covariates. These analyses were treated as robustness checks rather than as tests of substantive demographic hypotheses because the primary focus of the study was the relational model among emotional intelligence, self-regulation, and well-being.

Within the scope of the fourth research question, an indirect association analysis was conducted using Hayes’ PROCESS macro, Model 4, to examine whether emotional intelligence was indirectly associated with well-being through self-regulation (Hayes, 2022). In this model, emotional intelligence was specified as the independent variable, well-being as the outcome variable, and self-regulation as the variable through which the indirect association was estimated. The statistical support for the indirect association was evaluated using the bootstrap method with 5000 resamples and a 95% confidence interval. A bootstrap confidence interval that did not include zero was interpreted as indicating statistical support for the indirect association.

The indirect association model was first estimated without covariates and then re-estimated with the available demographic and family background covariates. The covariate-adjusted model included gender, nationality, number of siblings, family size, maternal education level, and paternal education level. Given the cross-sectional and correlational nature of the study, the indirect association estimates were evaluated not as causal effects but as statistical evidence of an indirect relational pattern among the variables. The significance level was set at 0.05 for all analyses.

## 3. Results

This section presents the findings in line with the research questions and hypotheses. First, descriptive statistics for emotional intelligence, self-regulation, and well-being are presented. Second, Pearson correlation coefficients are reported to examine the relationships among the variables. Third, simple and multiple linear regression analyses are presented to examine the variance in well-being accounted for by emotional intelligence and self-regulation when considered separately and jointly. Fourth, the indirect association between emotional intelligence and well-being through self-regulation is examined using PROCESS Model 4 with 5000 bootstrap resamples. Finally, covariate-adjusted robustness analyses are reported to examine whether the main findings remained stable after accounting for available demographic and family background variables.

### 3.1. Descriptive Statistics for the Variables

Descriptive statistics based on the item mean scores for the variables of emotional intelligence, self-regulation, and well-being examined in the study are presented in Table 1.

Table 1 presents the descriptive statistics based on item mean scores for the main variables of the study. Accordingly, the students’ mean emotional intelligence score was 3.06 (SD = 0.41). The mean self-regulation score was 2.33 (SD = 0.32), and the mean well-being score was 3.80 (SD = 0.65). These descriptive results address the first research question by summarizing students’ levels of emotional intelligence, self-regulation, and well-being.

### 3.2. Relationships Among the Variables

Pearson correlation analysis was conducted to determine the relationships among emotional intelligence, self-regulation, and well-being. The analysis results are presented in Table 2.

As shown in Table 2, there was a positive and significant relationship between emotional intelligence and well-being (r = 0.560, *p* < .001). There was also a positive and significant relationship between emotional intelligence and self-regulation (r = 0.407, *p* < .001). In addition, a positive and significant relationship was found between self-regulation and well-being (r = 0.293, *p* < .001). These findings supported H1, indicating positive associations among emotional intelligence, self-regulation, and well-being.

### 3.3. Regression-Based Associations with Well-Being

Simple and multiple linear regression analyses were conducted to examine the extent to which emotional intelligence and self-regulation accounted for variance in well-being. The analysis results are presented in Table 3.

In Model 1, emotional intelligence accounted for 31.4% of the variance in well-being, R^2^ = 0.314, F(1, 624) = 285.597, *p* < .001. Emotional intelligence showed a positive regression-based association with well-being, β = 0.560, t = 16.900, *p* < .001. In Model 2, self-regulation accounted for 8.6% of the variance in well-being, R^2^ = 0.086, F(1, 624) = 58.577, *p* < .001. Self-regulation showed a positive regression-based association with well-being, β = 0.293, t = 7.654, *p* < .001. In Model 3, emotional intelligence and self-regulation together accounted for 31.9% of the variance in well-being, R^2^ = 0.319, F(2, 623) = 145.957, *p* < .001. Emotional intelligence remained positively associated with well-being, β = 0.529, t = 14.608, *p* < .001. Self-regulation also remained positively associated with well-being, but its standardized coefficient was smaller, β = 0.078, t = 2.156, *p* = .031. The increase in explained variance from emotional intelligence alone, R^2^ = 0.314, to the joint model, R^2^ = 0.319, was very small. This indicates that self-regulation made a limited incremental contribution beyond emotional intelligence in this model. Overall, these findings supported H2.

### 3.4. Indirect Association Through Self-Regulation

An indirect association analysis was conducted using PROCESS Model 4 to examine whether emotional intelligence was indirectly associated with well-being through self-regulation. In the analysis, 5000 bootstrap resamples were used, and the statistical support for the indirect association was evaluated based on the 95% bootstrap confidence interval. The analysis results are presented in Table 4.

As shown in Table 4, emotional intelligence was positively associated with self-regulation, a path: B = 0.321, *p* < .001. Self-regulation was positively associated with well-being when emotional intelligence was included in the model, b path: B = 0.159, *p* = .031. The total association between emotional intelligence and well-being was statistically significant, c path: B = 0.902, *p* < .001. The direct association between emotional intelligence and well-being remained statistically significant when self-regulation was included in the model, c′ path: B = 0.851, *p* < .001. The indirect association between emotional intelligence and well-being through self-regulation was statistically supported, B = 0.051, boot SE = 0.024, 95% CI [0.005, 0.102]. Because the data were cross-sectional, this finding should be interpreted as a statistically supported indirect association rather than as evidence of temporal ordering or a causal mechanism. These findings provided statistical support for H3.

### 3.5. Covariate-Adjusted Robustness Analyses

Additional covariate-adjusted analyses were conducted to examine whether the main findings remained stable after accounting for available demographic and family background characteristics. Gender, nationality, number of siblings, family size, maternal education level, and paternal education level were included as covariates. The results of the covariate-adjusted regression model are presented in Table 5.

In the covariate-adjusted regression model, emotional intelligence and self-regulation remained positively associated with well-being after accounting for demographic and family background variables. The overall model was statistically significant, R^2^ = 0.334, adjusted R^2^ = 0.325, F(8, 606) = 37.95, *p* < .001. Emotional intelligence was positively associated with well-being, B = 0.791, SE = 0.059, β = 0.501, *p* < .001. Self-regulation also showed a positive but smaller association with well-being, B = 0.195, SE = 0.073, β = 0.097, *p* = .008. Among the covariates, only nationality showed a statistically significant association with well-being, whereas gender, number of siblings, family size, maternal education level, and paternal education level were not statistically significant. Overall, these results indicate that the main regression-based associations were substantively stable after the available demographic and family background variables were taken into account. The results of the covariate-adjusted indirect association model are presented in Table 6.

The covariate-adjusted indirect association model also supported the indirect association between emotional intelligence and well-being through self-regulation, B = 0.062, boot SE = 0.025, 95% CI [0.017, 0.114]. Because the bootstrap confidence interval did not include zero, the indirect association was statistically supported. Overall, the covariate-adjusted findings were consistent with the unadjusted model.

## 4. Discussion

This study examined the concurrent associations among emotional intelligence, self-regulation, and well-being in elementary school students within the same analytical framework. The findings showed a positive and statistically significant association between emotional intelligence and well-being (r = 0.560, *p* < .001), as well as between emotional intelligence and self-regulation (r = 0.407, *p* < .001). The association between self-regulation and well-being was also positive, but weaker (r = 0.293, *p* < .001). In the regression-based analyses, emotional intelligence accounted for 31.4% of the variance in well-being (R^2^ = 0.314), whereas self-regulation alone accounted for 8.6% (R^2^ = 0.086). When both variables were examined jointly, the model accounted for 31.9% of the variance in well-being (R^2^ = 0.319). In this model, emotional intelligence showed the stronger regression-based association with well-being (β = 0.529, *p* < .001), while self-regulation remained statistically significant but made a limited incremental contribution (β = 0.078, *p* = .031). The indirect association analysis indicated a small but statistically supported indirect association between emotional intelligence and well-being through self-regulation (B = 0.051, 95% CI [0.005, 0.102]). Because the data were cross-sectional, this finding should be interpreted as a statistically supported indirect association rather than as evidence of temporal ordering or a causal mechanism.

The covariate-adjusted robustness analyses further supported the main relational pattern. After gender, nationality, number of siblings, family size, maternal education level, and paternal education level were included as covariates, emotional intelligence and self-regulation remained positively associated with well-being. Emotional intelligence continued to show a stronger association, whereas self-regulation showed a smaller but still statistically significant association. The covariate-adjusted indirect association also remained statistically supported. These findings suggest that the main pattern of associations was substantively stable after the available demographic and family background variables were taken into account. Although nationality was statistically associated with well-being in the adjusted model, it was included as a control variable rather than as a substantive focus of the study; therefore, this finding should be interpreted cautiously and examined more directly in future research.

The finding that emotional intelligence was more strongly associated with well-being is consistent with previous research showing positive links between emotional intelligence and well-being in child and adolescent samples. Guerra-Bustamante et al. (2019) reported that adolescents’ emotional intelligence dimensions, particularly emotion understanding and regulation, were associated with perceived happiness. Similarly, the systematic review and meta-analysis by Llamas-Díaz et al. (2022) showed an overall positive relationship between emotional intelligence and subjective well-being among adolescents. Although these studies were conducted mostly with adolescent samples, they support the broader view that emotional intelligence is related to the well-being of school-aged individuals. The present study extends this line of evidence by showing that emotional intelligence is also meaningfully associated with well-being among fourth-grade elementary school students.

This finding should be interpreted in light of how emotional intelligence was measured in the present study. Emotional intelligence was assessed through a child self-report scale and was framed within a mixed-model-oriented understanding rather than as performance-based ability emotional intelligence. Therefore, the findings refer to children’s self-reported emotional intelligence, including emotional awareness, empathy, motivation, and managing emotions. This distinction is important because the observed association with well-being reflects children’s own reports of their emotional functioning rather than objectively tested emotional ability. Research on emotional intelligence in children indicates that emotional awareness, interpersonal relationships, school life, and socio-emotional functioning are closely connected to children’s developmental experiences (Özal et al., 2024). Gao et al. (2024) also showed that emotional intelligence was associated with elementary school students’ subjective well-being together with parenting styles and self-concept. Similarly, Pauletto et al. (2021) suggested that emotional self-perceptions in childhood are relevant to psychological well-being and developmental adjustment. Taken together, these findings support the interpretation that children who report stronger emotional intelligence also tend to report higher well-being.

The connection between emotional intelligence and broader school functioning also helps interpret the present findings. Herut et al. (2024) reported that emotional intelligence was associated with academic achievement among primary school students, while Nieto-Carracedo et al. (2024) showed that the relationship between emotional intelligence and academic achievement may be understood together with emotional well-being, motivation, and learning strategies. These studies are not identical to the present model; however, they suggest that emotional intelligence is linked not only to students’ emotional experiences but also to their motivation, learning processes, and school functioning. In this respect, emotional intelligence may represent a socio-emotional resource that is closely connected to children’s well-being in school life.

The finding that self-regulation was positively but more weakly associated with well-being should be interpreted carefully. The systematic review by Rodríguez et al. (2022) indicates that students’ self-regulation strategies are related to well-being, although this relationship may vary according to the dimension of well-being, sample characteristics, and measurement approach. In the present study, self-regulation accounted for a smaller proportion of variance in well-being than emotional intelligence, and its incremental contribution in the joint model was limited. This pattern suggests that self-regulation is relevant to well-being, but it should not be interpreted as an equally strong correlate in comparison with emotional intelligence in this sample. Self-regulation may be more directly related to learning behaviors such as goal setting, effort regulation, strategy use, help seeking, and self-evaluation. Well-being, by contrast, is a broader construct shaped by emotional, relational, and contextual factors as well as self-management skills. Therefore, self-regulation appears to function as a complementary developmental resource rather than as the central explanatory factor in the present model.

This interpretation is consistent with research on regulatory skills in childhood. Schlesier et al. (2019) showed that the school environment is an important context in which children’s regulation skills develop and can be observed. Olgun and Bulut Serin (2024) also examined emotion regulation in relation to children’s emotional and psychological well-being in the elementary school context and reported that some dimensions of regulation skills were associated with well-being scores. In the present study, self-regulation was statistically related to well-being, but the size of its contribution was modest. This finding indicates that self-regulation is relevant, yet its role should be interpreted in a measured way.

The indirect association findings further support the value of examining emotional intelligence, self-regulation, and well-being within the same model. The indirect association through self-regulation was statistically supported, but small in magnitude. This suggests that self-regulation accounted for a small part of the statistical association between emotional intelligence and well-being in this cross-sectional model, but this pattern does not constitute strong evidence of a causal or developmental mechanism. Studies on children’s well-being have similarly considered regulatory skills within indirect models. For example, Kılıç et al. (2024) examined child emotion regulation and regulation difficulties in relation to parenting and psychological well-being, while Gajda et al. (2025) investigated the role of self-regulation in the relationship between school stress and school well-being. These studies suggest that regulatory skills can be relevant within broader well-being models. However, the present findings indicate that, in this sample, self-regulation had a limited complementary role alongside the stronger association between emotional intelligence and well-being.

The findings also have cautious educational implications. They suggest that children’s well-being may be better understood when emotional and self-regulatory resources are considered together. However, the present study did not evaluate an intervention program and cannot show that improving emotional intelligence or self-regulation would lead to improvements in well-being. Therefore, educational implications should be framed as directions for future research and practice rather than as direct evidence of intervention effectiveness. In this respect, the findings are compatible with the broader literature on social-emotional learning. Cipriano et al. (2023), for example, showed that universal school-based social-emotional learning interventions can be associated with positive outcomes in students’ social-emotional skills, attitudes, behaviors, and school climate. Future longitudinal or intervention-based studies may examine whether classroom practices focusing on emotional awareness, emotion management, goal setting, self-evaluation, and help seeking contribute to children’s well-being over time.

### Limitations and Future Research Directions

The findings of this study should be interpreted in light of some limitations. The study used a cross-sectional and correlational design; therefore, the results indicate concurrent associations among emotional intelligence, self-regulation, and well-being but do not provide evidence of causal direction, temporal ordering, or developmental change. The statistically supported indirect association through self-regulation should also be interpreted within this limitation, as the design does not allow conclusions about developmental mechanisms. Future longitudinal, experimental, or quasi-experimental studies are needed to examine how emotional intelligence and self-regulation are related to children’s well-being over time. Another limitation concerns the use of student self-report scales. Self-report measures are useful for understanding children’s own perceptions, but they may be influenced by social desirability, item comprehension, attention span, and response tendencies. This is particularly relevant for emotional intelligence, which was assessed as self-reported emotional intelligence within a mixed-model-oriented framework rather than as performance-based ability emotional intelligence. Future studies may strengthen the evidence by using multi-informant and multi-method designs, including teacher assessments, parent reports, classroom observations, and performance-based measures.

The sample was limited to fourth-grade students from seven public elementary schools in Mersin, Türkiye. Although the schools were selected to reflect different district and school contexts, the findings should be generalized with caution to other age groups, grade levels, school types, and regional contexts. In addition, although demographic and family background variables were considered in covariate-adjusted analyses, other potentially relevant contextual variables, such as household income, parental involvement, school climate, teacher support, peer relationships, academic achievement, and classroom-level characteristics, were not included. Future research may use larger and more diverse samples and multilevel designs to examine how individual emotional and self-regulatory resources interact with classroom-, family-, and school-level factors. Finally, the indirect association through self-regulation was small in magnitude. Therefore, self-regulation should be interpreted as a complementary construct that showed a modest role in the statistical association between emotional intelligence and well-being rather than as a strong explanatory pattern. Future studies may examine additional indirect or moderating variables, such as emotion regulation, psychological resilience, academic motivation, school engagement, self-efficacy, social support, and family support, to develop more comprehensive models of children’s well-being.

## 5. Conclusions

This study examined the associations of children’s well-being with self-reported emotional intelligence and self-regulation among fourth-grade elementary school students. The findings showed that emotional intelligence was moderately and positively associated with well-being, whereas self-regulation showed a weaker but statistically significant association. The regression-based analyses indicated that emotional intelligence accounted for a larger proportion of variance in well-being, while self-regulation made a smaller but statistically significant incremental contribution to the model. The indirect association analysis also indicated a small but statistically supported indirect association between emotional intelligence and well-being through self-regulation.

These results suggest that emotional intelligence is an important socio-emotional correlate of children’s well-being during the elementary school years. In the present study, emotional intelligence was assessed through children’s self-reports and interpreted within a mixed-model-oriented framework, including emotional awareness, empathy, motivation, and emotion management. The findings therefore indicate that children who reported higher emotional intelligence also tended to report higher levels of well-being. At the same time, self-regulation appeared to function as a complementary self-management resource, showing a more limited association with well-being through processes such as goal setting, effort regulation, use of learning strategies, help seeking, and self-evaluation.

The findings may also inform future school-based research and practice. Rather than implying that emotional intelligence or self-regulation directly improves well-being, the results suggest that these developmental resources are relevant areas to consider when designing future longitudinal or intervention-based studies. In this respect, classroom practices that focus on emotional awareness, emotion management, empathy, learning responsibility, goal setting, and self-evaluation may be valuable topics for future empirical investigation. However, such implications should be interpreted cautiously because the present study did not evaluate an intervention program.

Overall, this study highlights the value of examining emotional intelligence and self-regulation together in relation to elementary school students’ well-being. Emotional intelligence emerged as the more central correlate of well-being in this sample, whereas self-regulation showed a smaller but meaningful complementary association. These findings indicate that children’s well-being can be understood more clearly when emotional and self-regulatory resources are considered together while avoiding causal conclusions that cannot be supported by a cross-sectional design.

## Figures and Tables

**Table 1:** Descriptive Statistics for the Variables.

Variable	*n*	M	SD	Min	Max
Emotional intelligence	627	3.06	0.41	1.56	4.00
Self-regulation	627	2.33	0.32	1.00	3.00
Well-being	626	3.80	0.65	1.42	5.00

Note. M = arithmetic mean; SD = standard deviation. Values are based on item mean scores for each scale.

**Table 2:** Pearson Correlation Coefficients among the Variables.

Variables	Emotional İntelligence	Well-Being	Self-Regulation
Emotional intelligence	1		
Well-being	0.560 **	1	
Self-regulation	0.407 **	0.293 **	1

Note. ** *p* < .001.

**Table 3:** Regression-Based Associations with Well-Being.

Model	Predictor Variable	B	SE	β	t	*p*	R^2^	F
Model 1	Constant	1.031	0.165		6.245	<.001	0.314	285.597
	Emotional intelligence	0.902	0.053	0.560	16.900	<.001		
Model 2	Constant	2.403	0.184		13.078	<.001	0.086	58.577
	Self-regulation	0.597	0.078	0.293	7.654	<.001		
Model 3	Constant	0.816	0.192		4.245	<.001	0.319	145.957
	Self-regulation	0.159	0.074	0.078	2.156	.031		
	Emotional intelligence	0.851	0.058	0.529	14.608	<.001		

Note. Outcome variable = well-being. SE = standard error. Model 1: F(1, 624) = 285.597, *p* < .001. Model 2: F(1, 624) = 58.577, *p* < .001. Model 3: F(2, 623) = 145.957, *p* < .001.

**Table 4:** Indirect Association between Emotional Intelligence and Well-Being through Self-Regulation.

Path/Effect Type	Relationship in the Model	B	SE	β	t	*p*	95% CI Lower	95% CI Upper
a path	Emotional intelligence → Self-regulation	0.321	0.029	0.407	11.161	<.001	0.265	0.378
b path	Self-regulation → Well-being	0.159	0.074	0.078	2.150	.031	0.014	0.304
Total association, c	Emotional intelligence → Well-being	0.902	0.057	0.560	15.784	<.001	0.790	1.015
Direct association, c′	Emotional intelligence → Well-being	0.851	0.065	0.529	13.099	<.001	0.724	0.979
Indirect association, a × b	Emotional intelligence → Self-regulation → Well-being	0.051	0.024	—	—	—	0.005	0.102

Note. SE = standard error; CI = confidence interval. The indirect association was evaluated using 5000 bootstrap resamples. A bootstrap confidence interval that did not include zero was interpreted as indicating statistical support for the indirect association.

**Table 5:** Covariate-Adjusted Regression Model for Well-Being.

Variable	B	SE	β	t	*p*	95% CI Lower	95% CI Upper
Emotional intelligence	0.791	0.059	0.501	13.382	<.001	0.675	0.907
Self-regulation	0.195	0.073	0.097	2.650	.008	0.050	0.339
Gender	0.072	0.043	0.057	1.669	.096	−0.013	0.157
Nationality	−0.262	0.068	−0.133	−3.873	<.001	−0.396	−0.129
Number of siblings	−0.016	0.040	−0.023	−0.392	.696	−0.095	0.063
Family size	0.032	0.051	0.036	0.625	.532	−0.068	0.131
Maternal education level	0.012	0.025	0.023	0.460	.645	−0.038	0.061
Paternal education level	0.001	0.024	0.002	0.050	.961	−0.047	0.049

Note. Outcome variable = well-being. N = 615. R^2^ = 0.334, adjusted R^2^ = 0.325, F(8, 606) = 37.95, *p* < .001. Gender, nationality, number of siblings, family size, maternal education level, and paternal education level were included as covariates.

**Table 6:** Covariate-Adjusted Indirect Association Model.

Path/Effect Type	Relationship in the Model	B	SE/Boot SE	t	*p*	95% CI Lower	95% CI Upper
a path	Emotional intelligence → Self-regulation	0.321	0.030	10.694	<.001	—	—
b path	Self-regulation → Well-being	0.195	0.073	2.650	.008	—	—
Total association, c	Emotional intelligence → Well-being	0.854	0.055	15.661	<.001	—	—
Direct association, c′	Emotional intelligence → Well-being	0.791	0.059	13.382	<.001	—	—
Indirect association, a × b	Emotional intelligence → Self-regulation → Well-being	0.062	0.025	—	—	0.017	0.114

Note. *n* = 615. The model included gender, nationality, number of siblings, family size, maternal education level, and paternal education level as covariates. The indirect association was evaluated using 5000 bootstrap resamples. Bootstrap confidence intervals are reported for the indirect association. “—” indicates that the statistic was not applicable or not estimated for that effect.

## Data Availability

The data that support the findings of this study are not publicly available due to confidentiality and ethics restrictions involving minors. The metadata codebook and analysis outputs may be made available from the corresponding author upon reasonable request, subject to institutional and ethical approval.

## Abbreviations

The following abbreviations are used in this manuscript:

EI	Emotional intelligence
SR	Self-regulation
WB	Well-being

## Appendix A

### Appendix A.1. Demographic Characteristics of Participants

**Table A1:** Demographic Characteristics of Participants.

Variable	Category	*n*	%
Gender	Girl	330	52.6
	Boy	297	47.4
Nationality	Turkish citizen	545	87.1
	Other	81	12.9
Number of siblings	Only child	64	10.3
	1 sibling	246	39.4
	2 siblings	179	28.7
	3 or more siblings	135	21.6
Family size	2 members	11	1.8
	3 members	54	8.8
	4 members	237	38.4
	5 or more members	315	51.1
Mother’s education level	Primary school	407	64.9
	Middle school	41	6.5
	High school	54	8.6
	University or above	125	19.9
Father’s education level	Primary school	417	66.5
	Middle school	27	4.3
	High school	56	8.9
	University or above	127	20.3
Mother’s occupation	Homemaker	378	63.3
	Public sector	118	19.8
	Private sector	68	11.4
	Self-employed	33	5.5
Father’s occupation	Unemployed	9	1.6
	Public sector	136	23.9
	Private sector	246	43.3
	Self-employed	177	31.2
Parental marital status	Married	550	88.3
	Divorced	61	9.8
	Deceased	12	1.9

Note. Percentages were calculated based on valid responses for each demographic variable; therefore, category totals may vary across variables due to nonresponse.

### Appendix A.2. Assumption, Diagnostic, and Robustness Checks

**Table A2:** Assumption and Diagnostic Checks.

Control	Value/Result	Interpretation
Emotional intelligence skewness	−0.325	Acceptable
Emotional intelligence kurtosis	−0.160	Acceptable
Self-regulation skewness	−0.536	Acceptable
Self-regulation kurtosis	0.457	Acceptable
Well-being skewness	−0.675	Acceptable
Well-being kurtosis	0.499	Acceptable
Tolerance	0.835	No multicollinearity problem
VIF	1.198	No multicollinearity problem
Condition Index	18.919	Acceptable
Durbin–Watson	1.949	Acceptable
Maximum Cook’s Distance	0.042	No influential observation

Note. Skewness and kurtosis values were within acceptable ranges, indicating that the variables showed distributional characteristics suitable for parametric analyses. Tolerance, VIF, and Condition Index values indicated no multicollinearity problem. The Durbin–Watson value was within an acceptable range. The maximum Cook’s Distance value was below 1, indicating that there were no influential observations likely to substantially affect the regression coefficients.

**Table A3:** Robustness Check for the Multiple Regression Model.

Variable	B	Conventional SE	HC3 SE	*p*	95% Bootstrap CI
Constant	0.816	0.192	0.195	<.001	[0.437, 1.199]
Emotional intelligence	0.851	0.058	0.065	<.001	[0.721, 0.976]
Self-regulation	0.159	0.074	0.074	.032	[0.017, 0.305]

Note. HC3 robust standard errors and 5000 resample bootstrap confidence intervals indicate that the direction and statistical significance of the findings in the main multiple regression model remained unchanged.
